# Chronic fatigue syndrome: Harvey and Wessely's (bio)psychosocial model versus a bio(psychosocial) model based on inflammatory and oxidative and nitrosative stress pathways

**DOI:** 10.1186/1741-7015-8-35

**Published:** 2010-06-15

**Authors:** Michael Maes, Frank NM Twisk

**Affiliations:** 1 Maes Clinics @ TRIA, Piyavate Hospital, Bangkok, Thailand; 2 ME-de-patiënten Foundation, Limmen, The Netherlands

## Abstract

**Background:**

In a recently published paper, Harvey and Wessely put forward a 'biopsychosocial' explanatory model for myalgic encephalomyelitis/chronic fatigue syndrome (ME/CFS), which is proposed to be applicable to (chronic) fatigue even when apparent medical causes are present.

**Methods:**

Here, we review the model proposed by Harvey and Wessely, which is the rationale for behaviourally oriented interventions, such as cognitive behaviour therapy (CBT) and graded exercise therapy (GET), and compare this model with a biological model, in which inflammatory, immune, oxidative and nitrosative (IO&NS) pathways are key elements.

**Discussion:**

Although human and animal studies have established that the pathophysiology of ME/CFS includes IO&NS pathways, these abnormalities are not included in the model proposed by Harvey and Wessely. Activation of IO&NS pathways is known to induce fatigue and somatic (F&S) symptoms and can be induced or maintained by viral and bacterial infections, physical and psychosocial stressors, or organic disorders such as (auto)immune disorders. Studies have shown that ME/CFS and major depression are both clinical manifestations of shared IO&NS pathways, and that both disorders can be discriminated by specific symptoms and unshared or differentiating pathways. Interventions with CBT/GET are potentially harmful for many patients with ME/CFS, since the underlying pathophysiological abnormalities may be intensified by physical stressors.

**Conclusions:**

In contrast to Harvey and Wessely's (bio)psychosocial model for ME/CFS a bio(psychosocial) model based upon IO&NS abnormalities is likely more appropriate to this complex disorder. In clinical practice, we suggest physicians should also explore the IO&NS pathophysiology by applying laboratory tests that examine the pathways involved.

## Background

**Figure 1:**
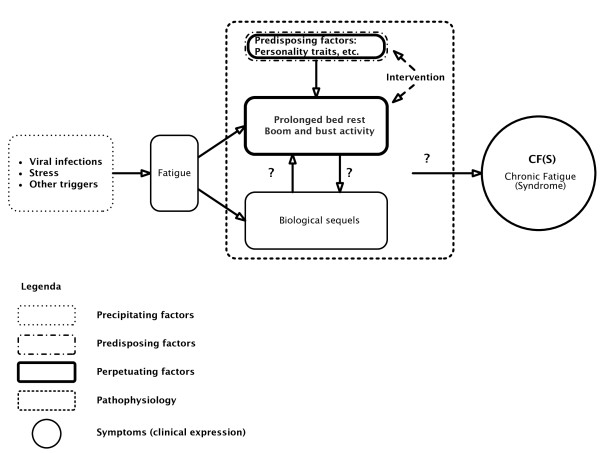
**The (bio)psychosocial model for 'chronic fatigue' of Harvey and Wessely**.

In a recent commentary, Harvey and Wessely [1] proposed a (bio)psychosocial model for all manifestations of chronic fatigue: myalgic encephalomyelitis/chronic fatigue syndrome (ME/CFS), unexplained fatigue, fatigue as a result of psychiatric conditions, and fatigue associated with an apparent medical cause, such as cancer, AIDS and autoimmune disorders. Figure 1 shows the Harvey and Wessely model [1].

In the view of Harvey and Wessely [1], stress, a viral infection or another trigger instigate 'fatigue' in predisposed individuals, which, mediated by prolonged bed rest, 'boom and bust activity' and biological sequelae (the maintaining factors), result in ME/CFS. The biological component of this model is restricted to the potential triggers (infections) and 'biological responses' to the initial fatigue, which, accompanied by 'behavioural responses' contribute to a prolonged severe fatigue. Perpetuating factors are principally behavioural ones; biological aberrations are considered to be a consequence not a cause. All predisposing factors, with one exception (childhood illness), are behavioural or characterological ones.

**Figure 2:**
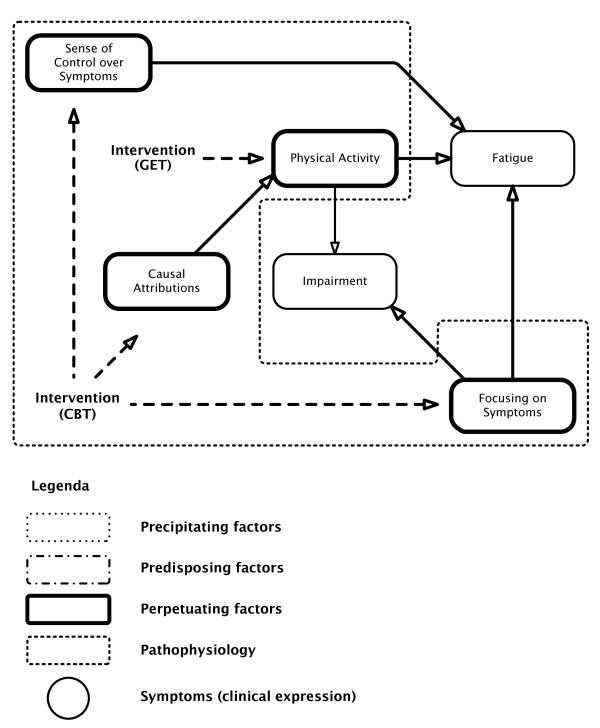
**The (bio)psychosocial model for myalgic encephalomyelitis/chronic fatigue syndrome (ME/CFS) of Vercoulen *et al*.** Fatigue: the subjective feeling of fatigue; fatigue subscale of the Checklist Individual Strength. Focusing on (Bodily) Symptoms: somatisation subscale of the Symptom Checklist. (Level of) Physical Activity: Sickness Impact Profile (SIP) subscale mobility (SIP-MOB) and the Physical Activities Rating Scale. (Functional) Impairment: impairment in daily life; subscale of activities at home of the SIP. Sense of Control (over Symptoms): selected items of the modified Pain Cognition List on a specific five-point scale. Causal Attributions: Causal Attributions List (high scores: physical attributions, low scores: psychosocial attributions).

Harvey and Wessely's [1] model strongly resembles the psychosocial model of Vercoulen *et al*. [2]. Figure 2 shows the Vercoulen *et al*. model. Fatigue and impairment are considered to be the end result of behavioural (psycho/sociogenic) factors only. According to this model attributing complaints to a somatic cause (physical attribution) negatively influences physical activity, which in turn has a negative impact on severity of fatigue and impairment. Focusing on symptoms also contributes to impairment and fatigue, and a low perceived sense of control over symptoms also induces fatigue. We will refer to both models as psychosocial models, since biological abnormalities are considered to play no role at all (the Vercoulen *et al*. model) or just a minor one (the Harvey and Wessely model) in explaining the symptomology of ME/CFS.

These psychosocial explanatory models for fatigue and ME/CFS and fatigue in general are the rationale for the combination of cognitive behavioural therapy (CBT) and graded exercise therapy (GET). In the biopsychosocial view, the patient can 'recover' by adjusting dysfunctional beliefs and behaviour and reversing deconditioning, which are proposed to be the maintaining factors in ME/CFS. CBT is aimed at eliminating psychogenic maintaining factors, for example illness beliefs, unhelpful, anxiety-provoking thoughts and kinesiophobia ('fear of movement'); CBT challenges the negative cognitions and dysfunctional beliefs of the patients [3]. CBT is indissolubly attached with GET, a rehabilitative approach of graded increase in activity to address deconditioning [3].

This paper examines Harvey and Wessely's [1] (bio)psychosocial model for ME/CFS and chronic fatigue in general and compares this model to a bio(psychosocial) model based on disorders in immune, inflammatory, oxidative and nitrosative stress (IO&NS) pathways.

## Comparison between the psychosocial models and a biological model based upon aberrations in IO&NS pathways

### The biological pathophysiology

**Figure 3:**
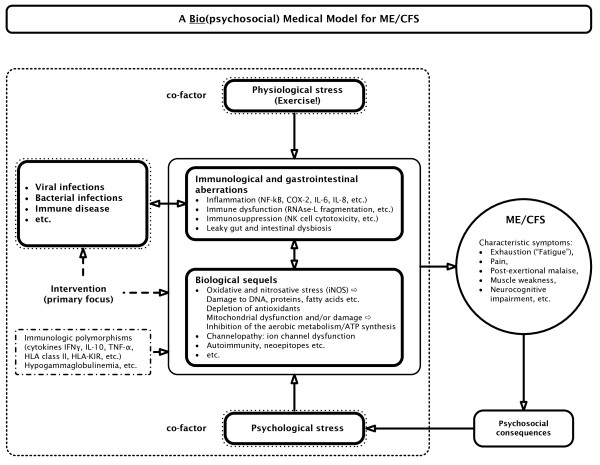
**The inflammatory and oxidative and nitrosative (IO&NS) pathophysiology of myalgic encephalomyelitis/chronic fatigue syndrome (ME/CFS)**. COX-2 = cyclo-oxygenase 2; iNOS = inducible nitric oxide synthase; PUFA = polyunsaturated fatty acids; NFκB = nuclear factor κB.

A medical model should explain how (a) precipitating and perpetuating factors induce (b) the biological pathophysiology that accounts for (c) specific symptoms. Figure 3 shows a biological model that plausibly explains 'fatigue' and ME/CFS by organic abnormalities and cause and effect relationships [4]. The model of Harvey and Wessely [1], however, does not specify (b) biological pathways that explain and maintain (c) the clinical picture and that are induced by (a) precipitating factors. Certainly, Harvey and Wessely [1] consider that a virus may trigger fatigue; however, their model does not include the concept that infections can trigger IO&NS pathways that may explain the consequent symptoms. We label the IO&NS-induced symptom complex 'fatigue and somatic' (F&S) symptoms, which encompass a flu-like malaise, fatigue, pain and muscle aches, cognitive disturbances, autonomic symptoms and so on [5]. Psychiatrists typically consider these symptoms to be 'functional' and label them as 'psychosomatic', 'hysteria' or 'somatisation'. Patients with specific F&S symptoms are diagnosed as ME/CFS patients once a number of diagnostic criteria are fulfilled (for example, as described by Fukuda *et al*. [6]). While Harvey and Wessely [1] acknowledge a minor role for biological factors as maintenance factors of ME/CFS, the cause and effect relationships of these biological factors are not specified.

However, there is evidence that IO&NS pathways play a key role in the pathophysiology of ME/CFS and other conditions in which F&S symptoms are prevalent. The findings that IO&NS pathways are involved in the pathophysiology of ME/CFS are replicated in many studies as reviewed previously ([4,7-9] and references therein). Figure 3 shows the pathways that are involved in F&S symptoms and ME/CFS. The factors involved are shown in Table 1[10-30].

**Table 1:** Overview of the different immune, inflammatory, oxidative and nitrosative stress (IO&NS) aberrations in myalgic encephalomyelitis/chronic fatigue syndrome (ME/CFS)

Aberrations in the IO&NS pathways in ME/CFS	Reference(s)
Increased production of key inflammatory mediators, such as NFκB, COX-2, iNOS	Maes *et al*. [10], Maes *et al*. [11]

Increased levels of proinflammatory cytokines	Fletcher *et al*. [12]

Immune activation, with increased *in vivo *expression of activation markers, such as CD38, and Th 1-like or Th 2-like responses	Klimas *et al*. [13]

Immunosuppression, for example, diminished natural killer cell activity (NKCA), and decreased ex vivo expression of activation markers, such as CD69	Maher *et al*. [14], Mihaylova *et al*. [8]

Depleted antioxidant levels	Maes *et al*. [15]

Increased levels of radical oxygen (ROS) and nitrogen species (RNS)	Kennedy *et al*. [16]

Oxidative damage to membrane fatty acids, mitochondria, functional proteins and DNA	Maes *et al*. [17], Behan *et al*. [18]

Autoimmune responses against oxidatively modified fatty acids and nitrated proteins (neoepitopes)	Maes *et al*. [9]

Autoimmune reactions	Maes (review) [19]

Gut dysbiosis and gut-derived inflammation with increased bacterial translocation	Maes *et al*. [20-22], Sheedy *et al*. [23]

Mitochondrial dysfunctions with lower carnitine and coenzyme Q10 levels	Myhill *et al*. [24], Plioplys and Plioplys [25], Maes *et al*. [7]

Upregulation and dysregulation of the 2'-5' oligoadenylate synthetase/RNase L pathway	Nijs and De Meirleir [26]

Apoptosis pathways	Gow *et al*. [27], Kerr *et al*. [28]

Ion channel dysfunction (channelopathy)	Broderick *et al*. [29]

Lowered omega-3 polyunsaturated fatty acids	Maes *et al*. [30]

For space considerations, this table only shows a selection of the relevant references. A more comprehensive listing can be supplied on request from the authors.

COX-2: cyclo-oxygenase 2; iNOS = inducible nitric oxide synthase; NFκB = nuclear factor κB; Th = T helper.

These, and other previously reviewed findings show that the F&S symptoms are a clinical expression of dysregulated IO&NS pathways [4]. As previously outlined [4], increased levels of proinflammatory cytokines, oxidative damage, increased cyclo-oxygenase 2 (COX-2) production, increased translocation by Gram-negative enterobacteria and so on, can generate F&S symptoms, including fatigue, a flu-like malaise, pain, symptoms of irritable bowel syndrome, and neurocognitive disorders. In addition, aberrations in IO&NS pathways are interrelated. For example, viral and bacterial infections and gut-derived inflammation may induce nuclear factor κB (NFκB) and consequently COX-2, inducible nitric oxide synthase (iNOS) and increased levels of proinflammatory cytokines. These inflammatory pathways and persistent or reactivating infections induce radical oxygen species (ROS) and radical nitrogen species (RNS), which in turn may damage membrane fatty acids, proteins, DNA and mitochondria. As a consequence of IO&NS, some cellular immune functions may be suppressed, for example, lowered natural killer cell activity (NKCA) and *ex vivo *expression of T cell activation markers, such as CD69. The aberrations mentioned above can also cause channelopathy, reduced omega-3 polyunsaturated fatty acid levels, cell death and apoptosis, and change inactive autoantigens into neoepitopes, which have acquired immunogenicity and act as triggers to bypass immunological tolerance. This process may explain the occurrence of an IgM-mediated autoimmune responses mounted against neoepitopes formed by O&NS damage to fatty acids, by oxidation, and proteins, by nitration. The above-mentioned processes may also explain the development of autoimmune responses against a plethora of self-antigens, including gangliosides and serotonin [4,20]. Depletion of antioxidants in patients with ME/CFS, partially due to inflammation, may further impair the protection against ROS and RNS, causing more damage to fatty acids, proteins, DNA and mitochondria [4,20]. Not every patient exhibits the full spectrum of these IO&NS disorders. In fact, each of the above-mentioned aberrations and/or combinations thereof may induce specific F&S symptoms, whereas ME/CFS is associated with the more severe forms of IO&NS aberrations (for example, severe inflammation, dysregulation of the RNase L pathway, apoptosis pathways, persistent damage to membrane lipids, functional proteins, DNA, and mitochondria and autoimmune responses [4,20]). For example, an initial infection may trigger the production of NFκB and consequently proinflammatory cytokines. These in turn may induce active immune-mediated symptoms (the F&S symptoms). Increased O&NS as a consequence of inflammatory responses can cause damage to membrane fatty acids, functional proteins, DNA or mitochondria, which further aggravate the immune-mediated symptoms. The primary inflammatory reactions can cause gut-derived inflammation that further aggravates inflammation and O&NS [10]. In some severe cases the above-mentioned processes may cause autoimmune responses against neoepitopes or self-antigens via, for example, mimicry [4,20].

It is often argued that the above-mentioned aberrations may be 'consequences' rather than the initial cause of the illness. However, as reviewed previously [4,8,9,20], there is ample evidence that immune activation and increased O&NS may induce F&S symptoms and, in specific cases, ME/CFS, and that treatments that target the IO&NS pathways may have a clinical efficacy in treating ME/CFS and F&S symptoms in general [4]. Moreover, these clinical findings are corroborated by observations in animal models demonstrating that experimentally induced activation of IO&NS pathways is accompanied by inflammation-induced 'fatigued' behaviour [31,32]. In this context, only a few examples are given. In mice, lipopolysaccharide (LPS)-induced peripheral immune activation is accompanied by fatigue. The severity of fatigue, motor deficits, coordination problems induced by immune activation correlate to the levels of the anti-inflammatory cytokine interleukin 10 [33]. In rats, intraperitoneal injections of a synthetic double-stranded RNA, polyriboinosinic:polyribocytidylic acid (poly I:C) induces profound fatigue, which is, amongst other things, characterised by increased levels of interferon-alpha that induces immune activation  [34]. In experimental animals, induction of O&NS by intensive and exhaustive exercise induces neurocognitive symptoms [35]. Evidence is also provided by animal models showing that experimentally induced F&S symptoms are associated with activation of the IO&NS pathways and that targeting IO&NS may reverse 'fatigued' behaviour [31-35].

It is often argued that 'animal models of ME/CFS' do not reflect ME/CFS in humans. However, translational research is of great importance in deciphering the pathways that may cause inflammation-mediated behavioural changes. Only one example is given: the effect of LPS. An increased immune response against the LPS from Gram-negative bacteria has been established in patients with ME/CFS, indicating a higher LPS load in their blood [20]. This may be the result of an increased translocation of Gram-negative enterobacteria from the gut to the blood due to increased gut permeability or leaky gut [20]. Animal experiments have demonstrated that LPS injected into the rodent may induce specific symptoms, such as F&S symptoms, through immune activation and neuroinflammation [20]. These translational experiments show that an increased immune response against LPS from Gram-negative bacteria, as has been detected in ME/CFS patients, can induce inflammation-mediated F&S symptoms [20].

### Aetiological factors

In humans, many studies have demonstrated that different pathogens can induce and/or maintain F&S and ME/CFS: viral (for example, Herpes Simplex, cytomegalovirus, Epstein Barr, Human Herpesvirus 6 (HHV-6)), and bacterial, (for example, *Chlamydia pneumoniae*, *Mycoplasma *species, enterobacteria, and *Coxiella burnetii *infections [4]). Psychosocial and physical stressors can be important precipitating and perpetuating (co)factors for F&S symptoms and gradual onset ME/CFS as well [4]. As reviewed previously, even moderate psychological stressors, such as examination stress, can induce the cytokine network [36] and O&NS pathways [4,37]. There is sufficient evidence that physical stressors activate the IO&NS pathways [4]. Harvey and Wessely [1] consider viral infections and stress to be triggers only, and don't consider their role in the pathophysiology of ME/CFS. Their model does not specify whether stress indicates physical or psychological stress. But more importantly, the model does not embody the important role of infections (viral and bacterial) and physical or psychological stress as cofactors. Therefore, we propose to include all above-mentioned precipitating factors in the biological explanatory model, as presented in Figure 3.

As stated by Harvey and Wessely [1], other factors can instigate ME/CFS symptoms (that is, the 'zebras', the rare organic causes of ME/CFS such as immune disorders). This is important because (auto)immune disorders, including autoimmune thyroid disorders, multiple sclerosis and rheumatoid arthritis, can induce F&S symptoms [19]. Consequently, '(auto)immune disorders' should be acknowledged as perpetuating factors for ME/CFS and F&S symptoms in general. In analogy to the diagnostic classification system in depression one could consider this concept as 'ME/CFS due to a general medical condition'. However, as discussed above, it has been demonstrated that patients with ME/CFS suffer from one or more IO&NS disorders and thus from an organic condition that can explain their F&S symptoms. An alternative view is that all the triggers mentioned above share the capacity to activate IO&NS pathways that eventually cause F&S symptomatology.

Harvey and Wessely [1] declare ME/CFS to be medically unexplained. However as stated above, the core elements of the organic pathophysiology of ME/CFS are already likely known: the induction of IO&NS pathways and its sequelae. As such the F&S symptoms of ME/CFS are largely explainable in terms of its pathophysiology. Of course, Harvey and Wessely [1] are correct to state that the trigger factors often remain unknown even after 'chasing the zebras'. Indeed, in the individual patient it not always possible to pinpoint the original trigger, because the trigger may have disappeared when the patient is examined. For example, a *Mycoplasma *infection may contribute to (chronic) activation of the IO&NS pathways, which eventually cause damage to lipids, proteins and DNA, gut-derived inflammation and autoimmunity [20]. The latter may persist after the *Mycoplasma *infection is eradicated by antibiotics [20]. Therefore, Harvey and Wessely's [1] discussion on whether the triggers (the zebras, the horses) can be pinpointed is in fact not really relevant, because ME/CFS can be understood by its pathophysiology even though sometimes (but not always) the precipitating factors cannot be pinpointed.

Based on their model, Harvey and Wessely [1] propose that the initial cause of the fatigue has a limited impact on the eventual course of the illness. This statement may be correct in some cases. However, it has been demonstrated that the severity of the illness and the rate of recovery of ME/CFS patients whose condition was triggered by specific infections may be determined by the acute phase of the infection [38,39]. More importantly, specific pathogens have been shown to cause persistent infections [40-43]. Whether or not the original trigger is still present and can be detected, the severity of F&S symptoms is significantly correlated to pathophysiological biomarkers. For example, Maes *et al*. [9-11,44] detected significant positive correlations between the severity of F&S symptoms in ME/CFS and IgM-mediated immune responses against neoepitopes originating from damage to O&NS-modified lipids and proteins, and increased NFκB, COX-2, and iNOS production. This indicates that even if the initial trigger has a limited impact on the course of the illness, there still is a significant association between the pathophysiological pathways induced by that trigger and the clinical manifestations of aberrations in the IO&NS pathways.

Harvey and Wessely [1] outline that even fatigue associated with 'apparent medical causes', such as cancer and HIV infection, is more closely associated with behavioural and psychological factors than with the severity of the underlying pathophysiology. However, in patients with autoimmune disorders, 'fatigue' can largely be explained by activated IO&NS pathways (for example, increased interleukin 1 (IL-1) [45]). In cancer patients, there is evidence that cytokines (for example, IL-6) play a key role in the fatigue [46]. HIV infection is characterised by fatigue accompanied by clinical signs of inflammation [47], an impaired quality of life that is related to immune activation [48], and a dysfunctional carnitine-dependent energy production [49].

### Predisposing factors

Harvey and Wessely's [1] model incorporates the assumption that some individuals are predisposed to develop fatigue and ME/CFS. They propose that personality factors, periodic overactivity, deconditioning, increased use of doctors, early childhood illness and so on, may predispose towards ME/CFS. However, central elements of their psychosocial model and the Vercoulen *et al*. model [2], including kinesiophobia and personality traits, have been disproved by various studies [50,51]. A recent study by Wiborg *et al*. [52], for example, invalidates the cause and effect relationship between physical activity and 'fatigue': improvement in the subjective feeling of 'fatigue' is not reflected by an increase in physical activity. The Vercoulen *et al*. model [2] as a whole has been invalidated by Song and Jason [50]. It is also interesting to note that in some studies, but not all, personality traits, coping mechanisms and psychiatric history do not seem to affect the outcome of CBT/GET, while immunological and related endocrinological variables do [51,53,54]. In addition, Harvey and Wessely [1] do not mention the immunological predisposing factors, such as polymorphisms in immune genes associated with ME/CFS [55-57], and deficiencies in immunoglobulins, such as IgG1, IgG2 and IgG3 [20,58].

### Maintaining factors

In the Harvey and Wessely model [1] biological factors are mentioned as maintaining factors. Harvey and Wessely [1], however, do not specify these 'biological factors'. There is no rationale to locate these pathophysiological maintaining factors between fatigue and CFS as Harvey and Wessely do. In our model, however, IO&NS abnormalities such as chronic inflammation, damage caused by IO&NS, and autoimmune disorders may persist, thereby becoming maintaining factors. The activated IO&NS pathways not only determine the expression of F&S symptoms, but can also determine the duration of those symptoms directly.

Other important maintaining factors in Harvey and Wessely's model are prolonged bed rest and boom and bust activity. Prolonged bed rest cannot be considered to be a maintaining factor, since often it is a dependent variable (not cause, but effect). As an illustration we refer to a case study [20]. This was a patient who suffered from ME/CFS, but was considered to be hysteric (la belle indifference) or psychosomatic by psychiatrists, the usual 'psychiatric' explanation. In this patient, a *Mycoplasma *infection induced an inflammatory cascade accompanied by O&NS-induced damage, a deficiency in specific antioxidants, gut-derived inflammation, autoimmunity to serotonin and gangliosides, resulting in paralysis and 'prolonged bed rest' [20]. In this case, one might expect the physician to conclude that the *Mycoplasma *infection is an important propulsive trigger factor for an IO&NS-driven pathophysiology, including severe inflammation and autoimmune responses, such as Guillain-Barre syndrome, with paralysis and, thus, prolonged bed rest [20]. It is, therefore, incomprehensible that Harvey and Wessely [1] consider a dependent variable to be an explanatory variable in their model, while the real explanatory variables (for example, the aberrations in the IO&NS pathways and their sequelae), and the bacterial infections, are not included.

How boom and bust activity may act as a maintaining factor in ME/CFS is not clear to us. Even if boom and bust activity would be relevant, its exact role in the model has yet to be determined and we would propose that this needs to be further verified by applying pathway analysis or other multivariate techniques in order to ascertain the significant cause and effect relationships between boom and bust activity and the IO&NS variables.

In addition, psychological stressors can be important maintaining factors for ME/CFS, as indicated by observations that acute and chronic psychological stress amplifies inflammation [16] and O&NS [37,59,60], which are already present in ME/CFS. Physical stressors can act as perpetuating factors for ME/CFS. This is substantiated by observations that exercise intensifies the pre-existing biological pathophysiology in many patients with ME/CFS [51].

### Symptomatology

In accordance with Jones *et al*. [61], Harvey and Wessely [1] propose to extend the definition of CFS by relaxing the exclusion criteria of the case definition of Fukuda [6]. Whether this is appropriate or not needs further investigation, we suggest by pattern recognition methods to determine whether specific patient clusters can be retrieved in a 'fatigued' population and to unravel the factor structure of F&S symptomatology. Unfortunately, in contrast to research in depression, to date not much effort has been put into multivariate studies in ME/CFS and fatigue in general. In some of our studies we have considered the differences in IO&NS pathways between patients fulfilling Fukuda's ME/CFS criteria and patients with chronic fatigue not fulfilling Fukuda's criteria. A distinction between ME/CFS and chronic fatigue is reflected by the type and severity of F&S symptoms, and by the biological abnormalities that are more prevalent in ME/CFS. Analyses of IgA responses against enterobacteria showed that these biological disorders are significantly more pronounced in ME/CFS than in chronic fatigue [21]. This situation is comparable to 'melancholic' depression, which is within the group of depressed patients (including minor and major depression) a qualitatively different category with regard to the severity and type of depressive symptoms and biological abnormalities [5].

Harvey and Wessely [1] suggest that the most common 'comorbid' condition in ME/CFS is depression, and they seem to suggest that if depression is present a mental state examination remains the best investigation. Depression frequently co-occurs with ME/CFS [62]. Because many patients with ME/CFS and related conditions have comorbid mood or anxiety disorders, it has been suggested that chronic fatigue is a 'form fruste' of depression [63]. Recently, we have shown that F&S symptoms are also key symptoms of (melancholic) depression. F&S symptoms observed in depression even seem to predict the severity and chronicity of depression [5]. Some patients presenting to physicians for evaluation of their symptoms concentrate on fatigue and are depressed. A mental state examination to identify patients with 'comorbid' depression is certainly warranted and treatment of depression, when present, is recommendable. The proposal of Harvey and Wessely [1] that a 'mental state examination' remains the best investigation in persons with 'unexplained fatigue' is not correct. First, as justified above, we would propose that analysing the aberrations in the IO&NS pathways is more important for unravelling the pathophysiology of ME/CFS and F&S symptoms in general than a mental state examination. Second, ME/CFS can be discriminated with a 100% accuracy from depression using percentage of time fatigue reported, severity of post-exertional malaise and shortness of breath, unrefreshing sleep severity, confusion/disorientation severity, and self-reproach [64]. In addition, a biological distinction between ME/CFS and depression has recently been confirmed by differential gene expression [65].

Further, we would suggest that the co-occurrence of depression, F&S symptoms and ME/CFS is more complex than suggested by Harvey and Wessely [1]. There is evidence that partially overlapping IO&NS pathways can induce two typical symptom clusters (that is, F&S symptoms such as pain, muscle tension, a flu-like malaise, neurocognitive complaints, and sleep disorders; and depressive complaints, such as sadness, loss of interest, psychomotor retardation, anorexia, weight loss and anhedonia [4,66,67]). For example, cytokine-based immunotherapy (with interferon-α) in patients infected with the hepatitis C virus induces two potentially coexistent symptom profiles: (a) F&S symptoms appearing early after starting treatment and occurring in almost all patients, and (b) depressive symptoms, occurring some weeks later in a subgroup of the patients [68,69]. Importantly, the severity of depression is even predicted by the severity of the F&S symptoms some weeks earlier [68]. In animal models, the above-mentioned F&S symptoms and depressive symptoms are induced by peripheral and central (neuro)inflammation, for example by interleukin 1β and tumour necrosis factor α (TNFα) [70,71]. This implies that depression and ME/CFS can be regarded as clinical manifestations of aberrations in shared IO&NS pathways. They can be distinguished by differences in other biological pathways (for example, the turnover of serotonin, neurodegeneration, decreased neurogenesis, and hypercortisolism, distinctive for depression [67]), and, for example, RNase L fragmentation [26,72] and hypocortisolism [73], characteristic for ME/CFS. Whether ME/CFS and depression are co-occurrent manifestations of shared IO&NS pathways requires further investigation.

### Therapy

When looking at the evidence base, it is not clear that the effectiveness of CBT/GET is significant in CFS/ME. The effectiveness of CBT/GET (20% to 40%), compared to support groups, natural course, standard medical care and so on (20% to 30%), is at best marginal [3]. If one takes into consideration the fact that self-rated fatigue was the only measure in most studies, 'fatigue' is a subjective measure that we suggest is largely insufficient to diagnose ME/CFS and has no correlation with objective measures, such as cardiopulmonary capacity [74] or physical activity [51]. Moreover, of the few randomised controlled trials considered to be relevant, almost all explicitly excluded large groups of ME/CFS patients and/or included non-ME/CFS patients, due to the selection criteria [51].

Previous papers have reported favourable effects for improvement in functional work capacity and fatigue by GET, irrespective of depression [75]. This could be explained by the fact that habitual exercise can improve autonomic nervous system adaptation and induce pulmonary and cardiovascular conditioning in a subset of chronic fatigued patients. However, in patients with ME/CFS, CBT/GET has been shown to be counterproductive in many patients. Based on the evaluation of the Belgian Reference Centres, the Belgian Minister of Health officially declared that CBT/GET should not be regarded as a curative therapy for ME/CFS [51,74]. This evaluation revealed that the exercise capacity/condition of the patients treated had not improved and that the occupational participation had even decreased after CBT/GET [51]. Two large-scale patient surveys in the UK and Norway, and two smaller surveys in Scotland and The Netherlands indicate that CBT/GET aggravates the condition of many ME/CFS patients [51]. It could be argued that GET may result in a 'late improvement' following 'initial worsening'. However, we have reviewed elsewhere that rehabilitation programmes, like CBT/GET, intensify characteristic F&S complaints, such as fatigue, pain, neurocognitive problems and so on [51]. This negative impact on the symptomatology of ME/CFS can be explained by the fact that exertion and GET may amplify the pre-existing pathophysiological aberrations, such as inflammation, O&NS; damage caused by O&NS, and sequelae such as mitochondrial dysfunctions and so on [51]. This suggests that the IO&NS pathways should be normalised before starting GET-like rehabilitation programmes. GET should, in our view, be accompanied by frequent assessment for IO&NS abnormalities. If present, they would strongly indicate that CBT/GET likely has no significant or even possibly negative effects. It could be argued, however, that the harm induced by CBT/GET will most likely occur in misdiagnosed patients, and it cannot be excluded that CBT may be a useful therapy for ME/CFS in some cases even in patients without major depression.

## Conclusions emerging from the IO&NS model presented here

Our comparison of Harvey and Wessely's model with a model more biologically based suggests that a biological model based upon IO&NS pathways is more appropriate to describe this complex organic disorder. Activation of the IO&NS pathways induces important characteristic ME/CFS symptoms, forms of chronic fatigue and F&S symptoms in general. The IO&NS pathways can be instigated by infections, viral and bacterial, psychosocial and physical stressors as well as medical disorders such as (auto)immune disorders, which all function as precipitating and/or maintaining factors for ME/CFS.

The IO&NS model presented here may explain the spontaneous improvement or resolution of illness that occurs in those with an acute onset and particularly in adolescents with an infectious onset. Spontaneous resolution of the symptoms may occur when these IO&NS responses are diminished once the initial infection is eradicated. However, in predisposed persons, the initial infection may induce extensive and long-term sequelae in the IO&NS pathways. Our pathophysiological model could also explain why F&S symptoms may persist in the absence of increased cytokine levels as is documented in post-infection fatigue studies [76]. The primary infections and subsequent responses in some specific cytokines might have resolved while the damage caused by O&NS to lipids, proteins or DNA and consequent autoimmune responses may persist and disable the patients, thus explaining residual F&S symptoms [19,20]. Moreover, it is always possible that neuroinflammation, other cytokines or proinflammatory products are involved which had not been measured in these studies.

It is important to note that the above-mentioned IO&NS pathways also offer a plausible explanation for the earlier mortality due to cardiovascular disorder in ME/CFS, which is described in detail elsewhere [77,78].

Based on their model, Harvey and Wessely [1] recommend clinicians to avoid spending too much time chasing 'rare or unlikely diagnoses', or in their own words: 'not to spend too much time looking for zebras among the horses', and they propose to limit the organic investigations to a small set of blood tests. In contrast, we suggest that clinicians should examine the IO&NS pathophysiology, their sequelae, and the possible precipitating and maintaining factors (for example, infections) in any given patient. In our opinion, not investigating the IO&NS pathways could lead to inappropriate diagnosis of the underlying pathophysiology and thus possible inappropriate treatment for the patient.

The set of blood tests proposed by Harvey and Wessely [1] includes some widely accepted tests for inflammatory diseases, such as C reactive protein and antibody assays for bacterial and viral infections and, in our opinion, should be conducted when assessing patients with ME/CFS. In addition, we suggest additional tests (outlined in Appendix 1) that are also good candidates and that can be performed as part of routine laboratory investigations; some of these are already approved by regulatory agencies. We think that regulatory agencies should objectively evaluate and approve the other tests, so that ME/CFS patients are reimbursed and these assays can be used on a regular basis in these patients.

Further, to identify the most accurate therapeutical approach it is likely necessary to define subtypes according to the IO&NS pathophysiology and the precipitating and predisposing factors [4,74]. Thus, a distinction should be made between the type of IO&NS disorders the patients suffer from. Does the individual patient suffer from gut-derived inflammation, T helper (Th) 1-like or Th 2-like immune responses, monocytic activation, dysregulation of RNase L pathway, channelopathy, mitochondrial dysfunction, a low coenzyme Q10 syndrome, damage to fatty acids, functional proteins or DNA, autoimmunity, and so on, or combinations of these aberrations? It is also important to uncover potential triggers and maintaining factors, such as bacterial and viral infections, psychological stressors, overexertion, and the rare 'zebras', such as (auto)immune disorders, that may explain the IO&NS pathophysiology of F&S symptoms and ME/CFS. Finally, it is important to pinpoint the predisposing and maintaining factors, such as IgG subclass deficiencies and immunosuppression with recurrent infections, respectively. When bacterial or viral infections have been shown to be important maintaining factors, antibiotics and antiviral medications are essential [4,74]. Intravenous immunoglobulins are an evidence-based treatment option for ME/CFS accompanied by common variable hypo-γ-globulinaemia or IgG subclass deficiencies, and recurrent infections or autoimmunity [20]. Treatment with carnitine, coenzyme Q10, and so on, may be advised in subjects with depleted mitochondrial functions [7,79]. Gut-derived inflammation responds favourably to treatment with glutamine [22]. Mouse models of ME/CFS demonstrate that F&S symptoms can be induced by peripheral and central IO&NS pathways, including lipid peroxidation and depleted antioxidant defences, and that those symptoms may be reversed by specific anti-inflammatory and antioxidant therapies targeting the IO&NS pathways [31,32]. Needless to say that there are still many IO&NS pathways in ME/CFS for which no adequate treatment exists, such as severe damage to lipids and proteins, and autoimmune responses.

We do not agree with the statement of Harvey and Wessely [1] that a mental state examination remains the best investigation in persons with 'unexplained fatigue' because the 'fatigue' could be explained by a mental condition, such as depression. First, because we would propose that a biological investigation is likely to be a better indication of the underlying causes that may account for many F&S complaints. Second, patients with ME/CFS can be discriminated from those with depression by using a characteristic symptom profile [64] or biological markers [72,73]. Third, as described above, the co-occurrence between depression, F&S symptoms and ME/CFS is complex. It appears that ME/CFS and major depression are symptomatic manifestations of shared IO&NS pathways. Based on the above, we propose that the F&S symptoms of ME/CFS are not caused by depression and that ME/CFS does not cause depression. This implies also that both disorders are distinct diagnostic categories that should be treated differently.

We propose that future research should use high throughput, high quality screening, as made possible by translational research employing genotyping microarrays and functional genomics (assays of IO&NS genes), novel IO&NS animal models of ME/CFS, including transgenic mouse models (IO&NS overexpression or knockouts), and promoter induction based indicator cell lines that are specific to the brain (for example, neuroinflammation, damage by O&NS), muscles (for example, damage by O&NS, mitochondrial dysfunctions) and the gut (for example, gut inflammation and gut-derived inflammation) in order to further delineate novel drug targets in the IO&NS pathways and develop new drugs to treat this complex medical disorder. Multivariate pattern recognition studies should be carried out in order to (a) define clinical subtypes of ME/CFS and chronic fatigue and their associations with co-occurrent depression, and (b) examine the shared IO&NS pathways versus those that discriminate both disorders. Finally, interventional studies should be carried out to test the clinical efficacy of (novel) drugs in treating the biological causes of ME/CFS subgroups, defined by biomarkers such as inflammatory profiles or gene expression.

## Competing interests

The authors declare that they have no competing interests.

## Authors' contributions

Both authors contributed equally to this review and reanalysis. The authors read and approved the final manuscript.

## Appendix 1

List of specific tests by category that should be carried out on a regular basis to investigate IO&NS abnormalities in ME/CFS.

### Inflammation

Proinflammatory cytokine tests: interleukin 1β (IL-1β), IL-6, and tumour necrosis factor α (TNFα).

T cell activation marker measurement by means of flow cytometry (for example, CD38+ T cells).

Anti-nuclear factor antibody tests.

Serotonin and ganglioside antibody tests.

Protein electrophoresis.

### Oxidative and nitrosative stress

IgM response tests against neoepitopes formed by O&NS damage to lipids and proteins.

Plasma carnitine tests (free, total and so on).

Plasma malondealdehyde (thiobarbituric acid reactive substances (TBARS)) test.

### Gut-derived inflammation

Tests to detect increased gut permeability.

### Predisposing factors

IgG subclass deficiency tests (IgG3 and so on).

## Pre-publication history

The pre-publication history for this paper can be accessed here:

http://www.biomedcentral.com/1741-7015/8/35/prepub
